# Dirac cone move and bandgap on/off switching of graphene superlattice

**DOI:** 10.1038/srep18869

**Published:** 2016-01-06

**Authors:** Tian-Tian Jia, Meng-Meng Zheng, Xin-Yu Fan, Yan Su, Shu-Juan Li, Hai-Ying Liu, Gang Chen, Yoshiyuki Kawazoe

**Affiliations:** 1 Laboratory of Advanced Materials Physics and Nanodevices, School of Physics and Technology, University of Jinan, Jinan, Shandong 250022, P. R. China; 2 Shandong Provincial Key Laboratory of Laser Polarization and Information Technology and Department of Physics, Qufu Normal University, Qufu, Shandong 273165, P. R. China; 3 New Industry Creation Hatchery Center, Tohoku University, Sendai, Miyagi 980-8577, Japan; 4 Kutateladze Institute of Thermophysics, Siberian Branch of Russian Academy of Sciences, Novosibirsk 630090, Russia

## Abstract

Using the density functional theory with generalized gradient approximation, we have studied in detail the cooperative effects of degenerate perturbation and uniaxial strain on bandgap opening in graphene. The uniaxial strain could split π bands into π_a_ and π_z_ bands with an energy interval E_s_ to move the Dirac cone. The inversion symmetry preserved antidot would then further split the π_a_ (π_z_) bands into π_a1_ (π_z1_) and π_a2_ (π_z2_) bands with an energy interval E_d_, which accounts for the bandgap opening in a kind of superlattices with Dirac cone being folded to Γ point. However, such antidot would not affect the semimetal nature of the other superlattices, showing a novel mechanism for bandstructure engineering as compared to the sublattice-equivalence breaking. For a superlattice with bandgap of ~E_d_ opened at Γ point, the E_s_ could be increased by strengthening strain to close the bandgap, suggesting a reversible switch between the high velocity properties of massless Fermions attributed to the linear dispersion relation around Dirac cone and the high on/off ratio properties associated with the sizable bandgap. Moreover, the gap width actually could be continuously tuned by controlling the strain, showing attractive application potentials.

Graphene consisting of hexagonally arranged carbon atoms has attracted tremendous research interests on both theory and experiment. Attributed to the *p*_z_ orbital forming π bonding states on both sides of graphene and the sublattice-equivalence confinement, the Dirac cone is formed by the valence band and conduction band crossing at K (K′) point. The linear energy dispersion relation around Dirac point brings us many unique and amazing properties, showing fascinating application potentials. The charge carriers, which could be tuned continuously between electrons and holes, have the group velocity as high as 10^6^ m/s being close to that of light. Therefore, the graphene-based nanostructures are believed to be promising materials for next-generation high-performance nanoelectronic devices^1^,^2^,^3^,^4^. However, since the conductivity due to the semimetal nature of graphene cannot be turned off completely, pristine graphene sheet cannot be used as a transistor in optoelectronics, where high on/off ratio is required. Recently, the methodology of quantum confinement by slicing graphene into nanoribbon, the substrate effects, the sublattice equivalence breaking, and the electric field effects are intensively investigated for the bandstructure engineering of graphene-based nanostructures^5^,^6^,^7^,^8^,^9^,^10^,^11^,^12^,^13^,^14^,^15^,^16^,^17^,^18^,^19^.

In the field of bandstructure engineering of graphene, an issue needs to be addressed. Along with the bandgap opening, the linear energy dispersion relationship around Dirac cone would be usually destroyed, which in turn would obviously reduce the charge carrier mobility. Usually, the wider bandgap opened in graphene to gain higher on/off ratio would lower the carrier mobility much more. Hence, it would be interesting if the bandgap could be switched on/off for a specific graphene-based nanomaterial toward different application demands. Interestingly, the advanced nanotechnologies have been successfully used in fabricating antidot-patterned graphene nanostructures–the graphene nanomeshes. The block copolymer lithography, nanoparticles local catalytic hydrogenation, nanosphere lithography, nanoimprint lithography, *etc*. have been proved to be efficient for synthesizing graphene nanomeshes^20^. In 2010, Bai *et al*. reported the nicely patterned graphene nanomesh fabricated by using the block copolymer lithography^21^. Recently, Wang *et al*. reported the CVD growth of large area smooth-edged graphene nanomesh by nanosphere lithography^22^. A most recent work^23^ on synthesizing large area graphene nanomesh tailored by interferometric lithography was reported by Kazemi *et al*. These progresses in fabricating advanced nanomaterials suggest the possibilities in preparing graphene nanomeshes in experiment with nice precision. In fact, this could be regarded as to pattern graphene into superlattice with carbon vacancies, which would modulate the corresponding Born-von Karman boundary conditions^24^,^25^,^26^. As shown in the experimental studies^20^,^21^,^22^,^23^, the antidot with circular vacancy hole may be easily realized, which preserves the inversion symmetry. Besides, the two dimensional superlattice may also be obtained through the interaction between sheet material and its supporting substrate, which could be seen in the two dimensional silicene grown on ZrB_2_ surface^27^. The periodic interaction pattern of silicene lattice in matching that of ZrB_2_ surface folds the Dirac cone to Γ point. So, to shed light on the experimental studies, we have carried out detailed studies of hexagonal-antidot-patterned graphene superlattice in which the inversion symmetry remains. The novel mechanism differing with the sublattice-equivalence breaking for bandstructure engineering and the effects of uniaxial strain on electronic properties are carefully discussed. Most interesting, for a kind of graphene superlattices whose bandgaps are opened at Γ point, the gap width could be continuously tuned by applying uniaxial strain until it is closed. So, for such superlattices, one could reversibly switch between the massless Fermion properties attributed to the linear dispersion relation around Dirac cone and the high on/off ratio properties associated with the sizable bandgap.

## Results and Discussion

### Energy band folding and bandgap opening

**Figure 1:**
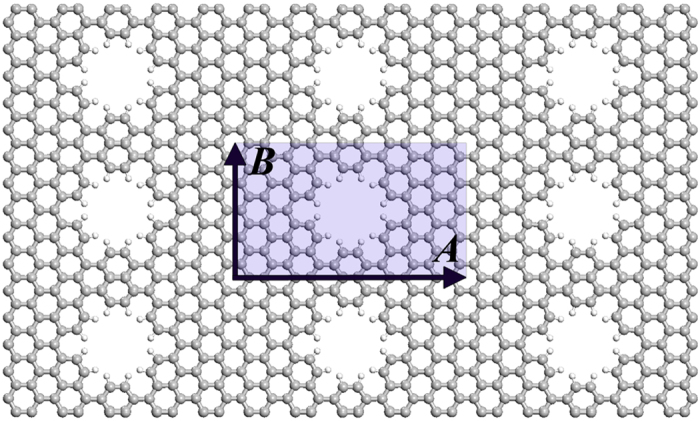
An anitdot-patterned orthogonal superlattice defined by ***A*** × ***B*** unit cell is schematically illustrated. The small and big balls are for H and C atoms, respectively.

The studied graphene superlattice is schematically illustrated in Fig. 1. The periodically arranged antidots impose new Born-von Karman boundary conditions to graphene, forming the regularly patterned superlattice. As shown in Fig. 1, we would like to concentrate on studying the superlattice patterned with *D*_6h_ high symmetry antidots in this paper for: (1) it could shed light on the circular mesh hole of the synthesized graphene nanomesh, and (2) it preserves the inversion symmetry. This kind of antidots in fact do not break the sublattice equivalence, which however could still open bandgap of some graphene superlattices, showing a novel mechanism for bandstructure engineering.

**Figure 2:**
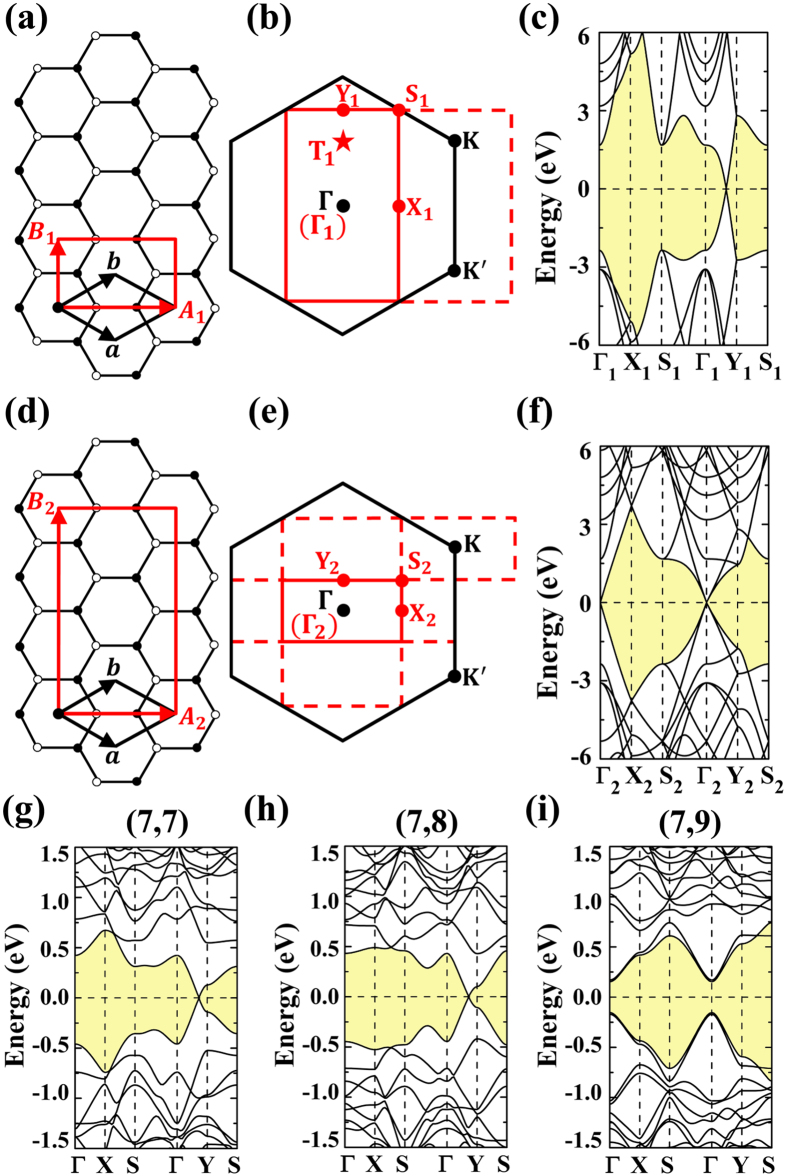
The schematic structure, Brillouin zone *r*-BZ, and energy bandstructure for the smallest orthogonal (1, 1) PGS are shown in (a–c), respectively. Those for the (1, 3) PGS are presented in (**d**–**f**), respectively. The Brillouin zone *h*-BZ corresponding to the primitive unit cell of graphene is also shown in (**b**,**e**) for comparison. The (**g**–**i**) are for the energy bandstructures of the antidot-patterned (7, 7), (7, 8) and (7, 9) graphene superlattices.

Firstly, we start the discussion of electronic properties with the energy band-folding analysis. In this paper, the orthogonal superlattice with the ***A*** × ***B*** periodic unit as shown in Fig. 1 has been carefully studied in detail. The ***A*** and ***B*** are along the armchair and zigzag edges, respectively. The smallest rectangle unit cell defined by ***A***_**1**_ = ***a*** + ***b*** and ***B***_**1**_ = −***a*** + ***b*** is illustrated in Fig. 2a along with the ***a*** × ***b*** primitive unit cell of graphene. As compared to the graphene studied with primitive unit cell, we would like to hereafter refer the pristine graphene studied with supercell ***A*** × ***B*** as pseudo graphene superlattice (PGS). If a perturbation such as an antidot was introduced in the ***A*** × ***B*** unit cell, the PGS would turn to be a real superlattice. In order to facilitate discussion, we would like to use the notation (*P*,*Q*) to account for the lattice ***A*** × ***B*** with ***A*** = *P**A***_**1**_ and ***B*** = *Q**B***_**1**_. Therefore, the (1,1) stands for the smallest rectangle lattice ***A***_**1**_ × ***B***_**1**_. In Fig. 2b, the hexagonal Brillouin zone (*h*-BZ) and the rectangle one (*r*-BZ) corresponding to the primitive unit cell and the (1,1) PGS are shown, respectively. One can see that the K point in *h*-BZ would be folded to the T_1_ 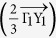 point in *r*-BZ. The energy bandstructure of (1,1) PGS is presented in Fig. 2c in which the Dirac cone is located at T_1_ point, confirming the above band-folding analysis. Based on our detailed analysis for the (*P*,*Q*) PGSes with different *P*, we could conclude that the Dirac point would be always folded onto 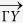 axis in the corresponding *r*-BZ. Interestingly, the Dirac point could be folded to Γ point when *Q* = 3*m* (*m* is an integer). In Fig. 2d–f, the (1,3) PGS is studied. The (1,3) unit cell is schematically shown in Fig. 2d and its corresponding *r*-BZ is presented in Fig. 2e. One can see that the K point in *h*-BZ of primitive unit cell is equivalent to the Γ_2_ point in *r*-BZ of (1,3) lattice. The energy bandstructure shown in Fig. 2f supports the folding of Dirac point to Γ_2_.

A bandgap can be opened in the (*P*,*Q*) (*Q* = 3*m*) PGS by introducing an inversion symmetry preserved defect, such as the *D*_6h_ hexagonal antidot, which does not coincide with the sublattice-equivalence breaking mechanism. For illustration, we have studied the electronic properties of (7,7), (7,8), and (7,9) superlattices. According to our energy band folding analysis, the (7,7), (7,8) PGSes should have Dirac points at 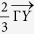 points in corresponding *r*-BZs while the (7,9) PGS should have Γ as Dirac point in its *r*-BZ. After introducing a C_12_ hexagonal antidot in the unit cell (In order to facilitate discussion, we would like to use the notation C_n_ to account for the antidot formed by removing a *n*-atom *D*_6h_ carbon nanoflake from the graphene with the hole edge passivated by hydrogen atoms), their bandstructures are studied and shown in Fig. 2g–i, respectively. Interesting it is the bandgap opening in the (7,9) superlattice while the (7,7) and (7,8) still keep semimetal conducting nature. Besides the orthogonal superlattices, we have also carried out careful studies on the other type superlattices. For a superlattice defined by ***A***_any_ × ***B***_any_, the lattice vectors could be expressed as ***A***_any_ = *N*_11_***a*** + *N*_12_***b*** and ***B***_any_ = *N*_21_***a*** + *N*_22_***b*** (***a*** and ***b*** are the basis vectors of primitive unit cell of graphene). If the coefficients satisfy the conditions of 2*N*_11_ + *N*_12_ = 3*m*_1_ and 2*N*_21_ + *N*_22_ = 3*m*_2_ or *N*_11_ + 2*N*_12_ = 3*m*_3_ and *N*_21_ + 2*N*_22_ = 3*m*_4_ (*m*_1_, *m*_2_, *m*_3_, and *m*_4_ are integers), the Dirac cone would be folded to Γ point and the introduced inversion symmetry preserved defect in the repeated unit cell would then open a bandgap in such superlattice.

Also, we would like to address the case of superlattice patterned with low symmetry antidots, where the inversion symmetry may be broken. The sublattice equivalence breaking would then open a bandgap in such superlattice. In fact, according to our calculated results, the novel bandgap opening mechanism proposed based the studies of inversion symmetry preserved superlattice would still stand, which would combine with the sublattice equivalence breaking mechanism in opening bandgap of the superlattice with Dirac cone being folded to Γ point. As a result, the mass bandgap would be determined by the dominated mechanism which could open a wider gap.

### Splitting between π_a_ and π_z_ bands

In the above discussion, the hexagonal antidot acts differently on bandgap engineering of the free-standing graphene superlattice, which strongly depends on the position of the Dirac point. Only for the lattice with *Q* = 3*m*, the antidot could open bandgap. Actually, the Dirac cone could be moved away Γ point by some perturbation, such as the uniaxial strain. One may question whether the opened bandgap of antidot-patterned (*P*,*Q*) superlattice with *Q* = 3*m* could be closed by moving Dirac cone away Γ point ? Hence, in this subsection, we would like firstly to concentrate on discussing the effects of uniaxial strain on the Dirac cone move.

**Figure 3:**
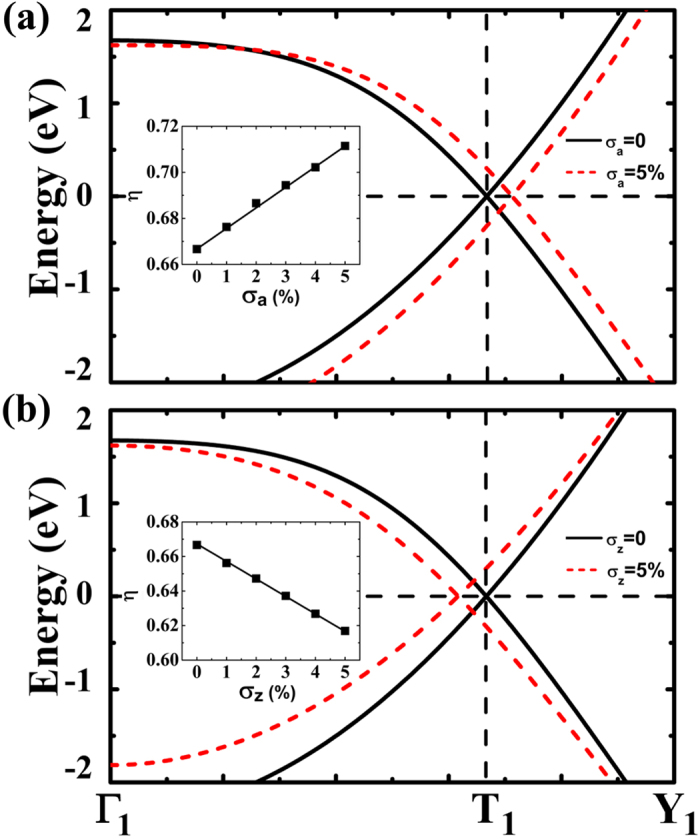
The shifts of Dirac point along ΓY of the reciprocal lattice for the graphene under 5% uniaxial stretching strain applied along armchair (a) and zigzag (b) edges, respectively. The insets show the deviation of Dirac point referred to its position in free-standing graphene as a function of the applied strain. Only π bands those cross to form Dirac point are shown for clarity. The notations of *k*-points Γ_1_, T_1_, and Y_1_ are illustrated in Fig. 2b.

In Fig. 3, as to the smallest rectangle (1,1) PGS, we have carefully studied the energy bandstructures after applying uniaxial strain along the armchair (***A***_**1**_ lattice) and zigzag (***B***_**1**_ lattice) edges, respectively. In our studies, the Dirac cone was found to move along the 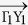 axis. In the free-standing PGS without strain, the Dirac cone is located at 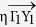 with 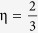 (marked with T_1_ point in Fig. 2). The strain σ_a_ applied along the armchair edge would move Dirac cone away T_1_ point with 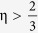 while the strain σ_z_ along the zigzag edge would move it toward Γ point 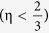.

**Figure 4:**
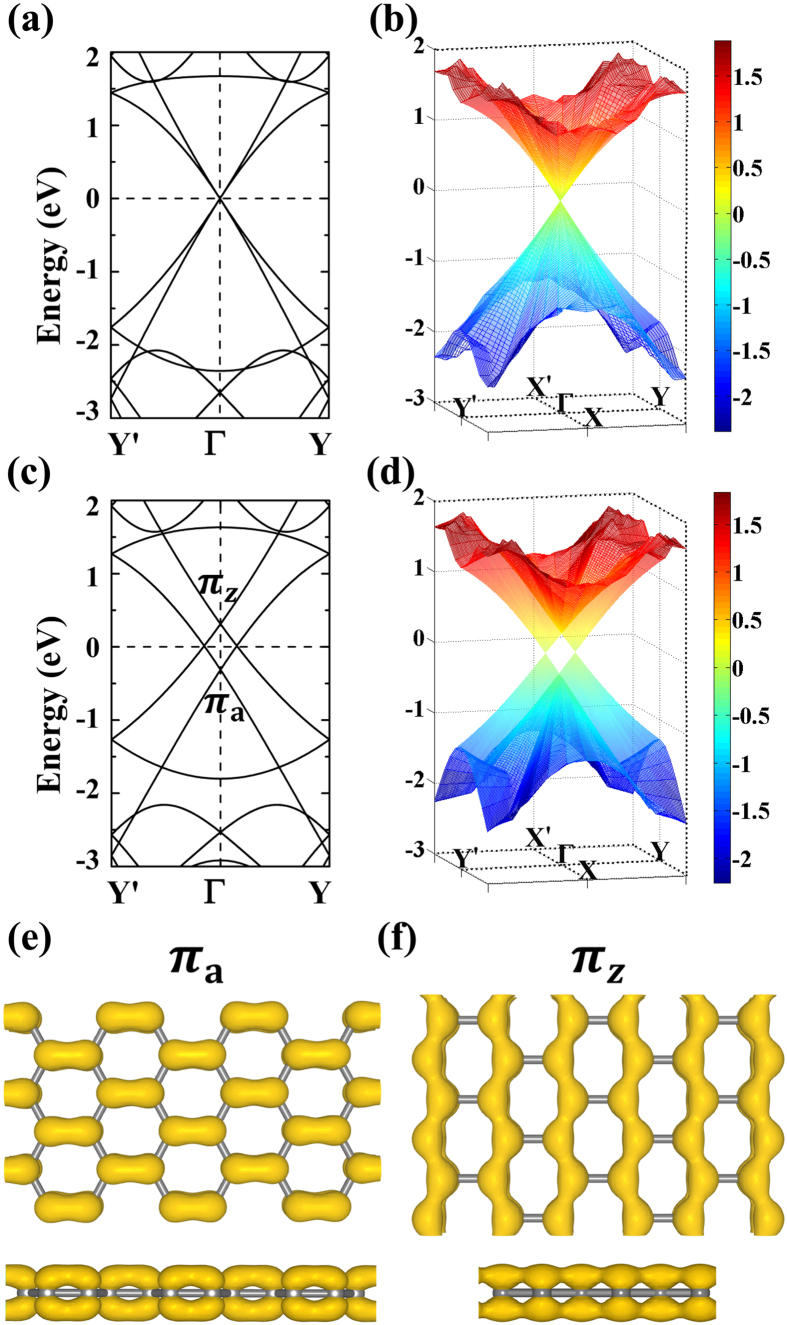
The band-folded energy bandstructure along Y′-Γ-Y path in reciprocal space (a) and the three-dimensional plotting of the corresponding Dirac cone (b) for the free-standing (3, 3) PGS. Those for the (3, 3) PGS under 5% σ_z_ strain are shown in (**c**,**d**), respectively. The band-decomposed charge densities at isovalue of ~0.03 e/Å^3^ for the split π_a_ and π_z_ bands are presented in (**e**,**f**), respectively. Both top and side views are shown.

In order to investigate the effects of Dirac cone move on the bandgap opening of the *D*_6h_-defect-patterned superlattice, the (3,3) PGS lattice is adopt as a prototype example. As shown in Fig. 4a, the band closing point of (3,3) PGS is folded to Γ point, which corresponds to the Dirac cone shown in Fig. 4b. In our studies, the effects of the strains σ_a_ and σ_z_ are investigated in detail. Both of them could shift the Dirac cone away Γ. In Fig. 4c, the effects of the strain σ_z_ are illustrated. The band closing point at Γ is degenerate in Fig. 4a, which is now split by the strain σ_z_ as shown in Fig. 4c. The Fig. 4d shows the three dimensional plotting of Dirac cones, confirming their moves along 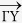 and 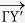 respectively. In Fig. 4c, two band crossing points at Γ appear now, which are marked as π_z_ and π_a_ respectively. The charge densities of the π_z_ and π_a_ bands are plotted in Fig. 4e,f, respectively. One can see that the π_z_ band accounts for the orbitals along zigzag edge while the π_a_ is for the orbitals along armchair edge. This suggests that the Dirac point move could be attributed to the splitting between the energy degenerate bands π_z_ and π_a_. Interesting it is that the density distributions of π_a_ and π_z_ bands show anisotropic characters. However, due to the fact that they would cross with each other at Fermi level in the neighborhood of Γ point, the anisotropic characters could not be observed in the conducting property studies. But, as to be discussed, the peculiar phenomena may be obtained in the strain engineered defect-patterned suplattice. For the (3,3) PGS without strain, the bond lengths of π_z_ and π_a_ bonds are exactly the same, which are around 1.425 Å. By applying the 5% σ_z_ strain along zigzag edge, the π_z_ bond is elongated by ~3% while the π_a_ bond remains almost unchanged. The tight binding (TB) method is well known in treating π bands of graphene, which could help to understand the physical origin of the electronic properties of graphene-based nanostructures. By considering only the nearest neighbor atoms, the related energy E_nna_ could be obtained through^28^
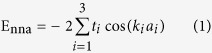

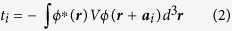
where, *t*_*i*_ is the transfer integral, *k*_*i*_ is the component of wavevector ***k***, *a*_i_ is the corresponding lattice constant, and 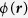 is the atomic orbital. According to TB method^28^, the ~3% elongation of C-C bond length suggests the weakening of the corresponding transfer integral, resulting in the energy band lift of zigzag edge bond in Fig. 4c. The crossings of π_z_ and π_a_ orbitals at Fermi level make two separated Dirac points adjacent to Γ. In the spirit of the concept of pseudospin, two valleys with different chiralities of the corresponding electrons are located at K and K′ points in the *h*-BZ of primitive unit cell of graphene. In the pseudo superlattice (3,3), the valleys are folded onto Γ point in the corresponding *r*-BZ. The effects of the uniaxial strain are to split the degenerate valleys by shifting them away Γ in opposite directions, which would still keep them equivalent in energy with the apexes of corresponding Dirac cones being located at Fermi level.

### Splitting between π_a1_ (π_z1_) and π_a2_ (π_z2_) bands

On a hand, due to the linear dispersion relationship around Dirac cone, the charge carriers gain high group velocity, which could also be tuned between the holes and electrons. On the other hand, the semimetal nature of graphene does not favor its usage as for high-performance nano-transistors, which requires high on/off ratio. In order to obtain high on/off ratio, a bandgap needs to be opened for the graphene-based nanostructure. However, along with the bandgap opening, the linear dispersion relation of graphene crystal would be destroyed also, which in turn harms the velocity properties of the charge carriers. So, it would be interesting to explore the possibilities in switching between the high velocity properties of massless Fermions and the high on/off ratio properties. The *D*_6h_-antidot-patterned superlattice studied in this paper may be a potential candidate. In (*P*,*Q*) (*Q* = 3*m*) PGS, the Dirac point would be folded to Γ point. Then, the high symmetry *D*_6h_ antidot can open a bandgap in the corresponding graphene superlattice, which however could not change the semimetal nature of the other superlattices with *Q*≠3*m* (the Dirac point is not at Γ). Interestingly, by controlling strain, one can manipulate whether the Dirac point is located at Γ point or not of the (*P*,*Q*) (*Q* = 3*m*) PGS, which suggests the possibilities in switching on/off bandgap of such superlattice.

**Figure 5:**
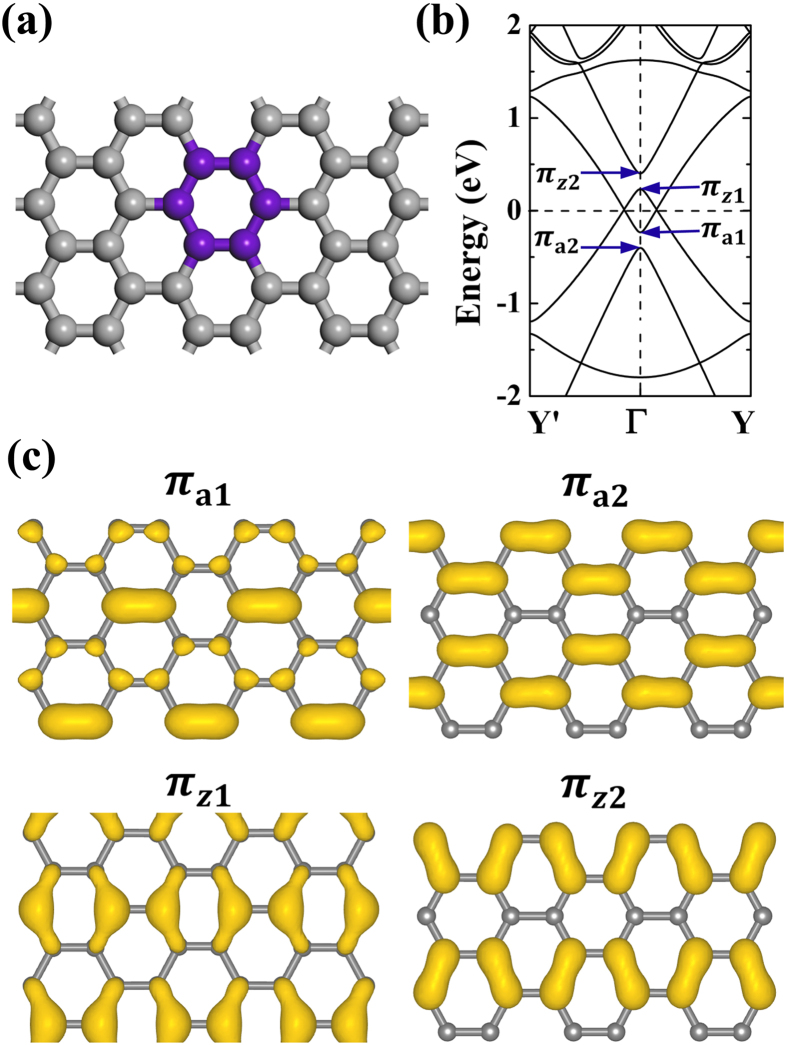
The *D*_6h_ defect formed by contracting the C-C bonds by 3% of the black (purple online) hexagon (a) and the corresponding bandstructure along the Y′-Γ-Y path for the (3, 3) under 5% σ_z_ strain (b). The corresponding band-decomposed charge densities at isovalue of ~0.02 e/Å^3^ for the split π_a1_, π_a2_, π_z1_, and π_z2_ bands are shown in (**c**).

As to the (3,3) PGS, in order to facilitate discussion, we would like to first study the effects of a defect in the unit cell by contracting a C_6_ hexagon (see the structural illustration in Fig. 5a). The C-C bonds in this hexagon are shortened by 3% to enhance the corresponding transfer integral^28^. The atoms of this C_6_ hexagon are fixed while the rest atoms are allowed to relax. For the free-standing (3,3) PGS, the introduced defect could open a bandgap at Γ point according to the discussion of the bandgap opening mechanism. Applying a strain σ_z_ of ~5%, we have then studied its electronic properties again. The corresponding bandstructure along Y′-Γ-Y path is shown in Fig. 5b. Now, the bandgap gets closed with the closure Dirac points being around Γ, realizing the bandgap switching off.

Compared to the electronic properties shown in Fig. 4c for the (3,3) PGS under 5% σ_z_ strain, where the strain makes the splitting between π_z_ and π_a_ orbitals, the *D*_6h_ defect also induce band splitting. As shown in Fig. 5b, the split bands are marked with π_a1_, π_a2_, π_z1_, and π_z2_, respectively, for which the corresponding band-decomposed charge densities are studied in Fig. 5c. It is obvious that the *D*_6h_ defect breaks the equivalence among π_a_ (π_z_) orbitals, resulting in different bonding strengthes between π_a1_ (π_z1_) and π_a2_ (π_z2_) bonds. As compared to the corresponding C-C bonds of (3,3) PGS studied in Fig. 4c, the contraction of the C_6_ hexagon induces about 0.8% elongation of π_a1_ bonds and 0.6% contraction of π_a2_ bonds, respectively. As to the π_z1_ and π_z2_ bonds, the corresponding elongation and contraction are about 1.0% and 0.7%, respectively. In the spirit of the TB method^28^, the shorter the bond is, the higher the transfer integral is, and then the lower the band energy is. Accordingly, as shown in Fig. 5b, the π_a2_ and π_z1_ bands are lower in energy compared to the corresponding π_a1_ and π_z2_ bands, accounting for the splitting of π_a_ and π_z_ bands. Actually, the herein discussed splitting could also be understood by the intervalley scattering. The periodically arranged defects could be approximately treated as introducing a periodic potential V_d_(***r***) to the pristine grephene. If the wavervectors of the opposite ***K*** and ***K ***^′^ valleys differ with each other by a reciprocal lattice ***G***, the intervalley scattering would induce band splitting. The energy interval E_d_ would be proportional to the integration 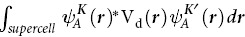 with 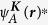 and 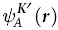 as the Bloch functions of sublattice A at ***K*** and ***K***^ ′^ points^29^.

### Reversible on/off switching of bandgap

Now, one can see that (1) the strain along either armchair or zigzag edges could make the degenerate π bands to split into π_a_ and π_z_ bands and (2) the defect could break the equivalence among the armchair edge bonds (zigzag edge bonds) inducing the band splitting also. In fact, the competition between the band-split interval E_s_ induced by the uniaxial strain and the E_d_ induced by the introduced defect dominates whether the bandgap could be switched on or off. Actually, we have also examined the free-standing defect-patterned (3,3) superlattice. The studied *D*_6h_ defect also induces the band split E_d_ between π_a1_ (π_z1_) and π_a2_ (π_z2_) orbitals. For there is no strain applied to the lattice, the corresponding E_s_ equals to zero. So, an obvious bandgap could be opened at Γ point. However, for the (3,3) superlattice studied in Fig. 5b, the E_s_ is larger than the E_d_, which makes the π_a1_ and π_z1_ to cross to form new Dirac cone.

**Figure 6:**
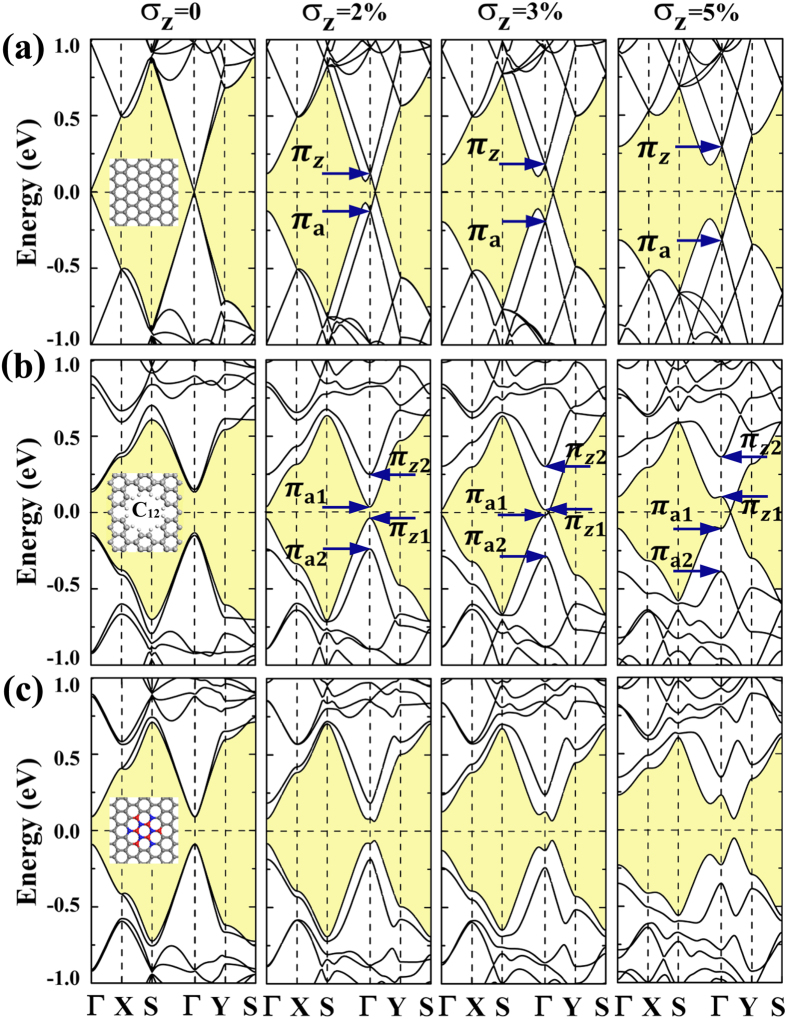
The calculated energy bandstructures for the graphene-based nanostructures under 2%, 3%, and 5% σ_z_ strains and the ones for the free-standing materials. The (**a**–**c**) are for the (8, 9) PGS, C_12_-patterned superlattice, and (BN)_6_-patterned superlattice, respectively.

In order to benefit experimental studies of patterning graphene nanomesh with circular antidots, we have also studied the bandgap on/off switching of the *D*_6h_-antidot-patterned graphene superlattice by applying strain. In Fig. 6, the (8,9) PGS is used for discussion. The corresponding graphene sheet has been calculated by employing the (8,9) supercell instead of the primitive unit cell, which would be used for comparison with the defect patterned superlattices. For the free-standing PGS lattice, the Dirac cone is folded to Γ point (see the panel of σ_z_ = 0 in Fig. 6a). After applying strain, the degenerate π bands begin to split. The corresponding energy interval E_s_ increases as the strain σ_z_ enhances from 0% to 5%. As shown in Fig. 6b, the C_12_ antidot patterned superlattice has been studied. Besides of the experimental fact of the successful fabrication of vacancy hole patterned graphene superlattice, we have performed the molecular dynamics simulations to evaluate the stability of the C_12_ patterned superlattice. We find that it can withstand the temperature of 1,500 K, implying that this superlattice structure is separated by high-energy barriers from other local minima on the potential energy surface. In this superlattice, the C_12_ antidot further splits the π_a_ and π_z_ orbitals, which brings the E_d_ ≈ 0.28 eV energy interval. For the studied (8,9) superlattice, the E_d_ remains almost the same as the applied strain increases. For the σ_z_ = 2% strain, the E_s_ is smaller than the E_d_. This still keeps the bandgap opening though the bandgap becomes narrower. An interesting point needs to be addressed. Now, the conduction band minimum (CBM) and the valence band maximum (VBM) consist of only π_a_- and π_z_-type bands, respectively, hinting the possibilities in obtaining anisotropic conducting properties. The electron and hole charge carriers may respectively transport along armchair and zigzag directions. However, for the case of the σ_z_ = 3% strain, the split energy interval E_s_ gets larger than the E_d_. The Dirac cone starts to form. For the condition with σ_z_ = 5% strain, the formation of the Dirac cone is obvious. So, for the energy interval E_d_ induced by the defect keeps almost constant, one could then switch on/off the bandgap by manipulating the energy interval E_s_ through controlling uniaxial strain. This would be interesting for that the high velocity properties of massless Fermions attributed to the linear energy dispersion around Dirac cone and the high on/off ratio properties associated with the sizable bandgap could be switched with each other in the same material. For comparison, the superlattice patterned with (BN)_6_ flake (patching the C_12_-antidot hole with this nanoflake) has also been studied. For the graphene and BN sheet materials, the atoms are correspondingly hexagonally arranged in a plane to form the honeycomb structures. The calculated lattice constants of graphene and BN sheets are 2.47 and 2.51 Å, respectively. By using (BN)_6_ nanoflake to patch the vacancy hole in graphene, the local strain around the nanoflake should stand due to the lattice mismatch. Considering the fact that the ratio of the area of nanoflake against the one for the graphene is only around 0.04, the local strain would probably be released to some sense at the aid of the structural relaxation of graphene, which would help to stabilize the patched superlattice structure. In order to evaluate the structural stability, we have also carried out the MD simulations of the (BN)_6_ patched (8,9) superlattice, which suggest the stability at temperature of 1,500 K.

In Fig. 6c, the calculated energy bandstructures always show semiconducting properties. Here, the bandgap opening mechanism should be different as compared to that of the C_12_-antidot-patterned superlattice. The (BN)_6_ flake breaks the inversion symmetry by destroying the sublattice equivalence, opening ~0.13 eV bandgap. This bandgap seems unaffected in gap width by the uniaixal strain in our studies. Also, the deformed bandstructures induced by the uniaxial strain presented in Fig. 6c hint that the (BN)_6_ patched superlattice may have topological semiconducting properties due to the anticrossing bands shown in the neighborhood of Γ point, which are worthy of further investigations on both theory and experiment. As discussed the above, the uniaxial stretching strain would modify the electronic properties accordingly. Because of the linear dispersion relation around Dirac cone, the group velocity of massless Fermions could be estimated by the band energy gradient (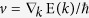)^28^. The *k* and E(*k*) are the wave vector and dispersion relation, respectively. For the fact that the mobilities of the charge carrier electrons and holes are almost same, we would like to concentrate on discussing their average values, with the purpose to illustrate the effects of strain. The average velocities of the PGSes studied in Fig. 6a are calculated and presented in Table 1, which are around 8.24, 8.15, 8.07, and 7.90 × 10^5^ m/s for the corresponding graphene superlattices under 0%, 2%, 3% and 5% strains, agreeing with the experimentally measured Fermi velocity of ~10^6^ m/s for graphene^2^,^4^. For the semiconducting graphene superlattice, the linear dispersion relation is destroyed, which now could be described by parabola function around the band extremum. Thus, the corresponding effective mass could be obtained by^28^
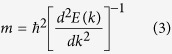


In Table 1, we present the calculated average values. Starting from the free-standing C_12_-antidot-patterned (8,9) superlattice, the opened bandgap would be continuously reduced by enhancing strain, and the corresponding effective mass would be decreased also. According to the simple relationship 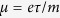 between carrier mobility (*μ*) and effective mass (*m*) (*τ* is the scattering time of charge carrier)^28^, the smaller the bandgap is , the higher the charge carrier mobility would be. By using the rough estimation of 10^−13^ s for scattering time, we have also estimated the carrier mobilities of the semiconducting superlattices to be ~10^3^ cm^2^V^−1^s^−1^. Once the bandgap starts to close, the band energy dispersion would gain linear relationship again, resulting in high charge carrier mobility. For the case studied in Fig. 6b under σ_z_ = 5% strain, the group velocity of the massless Fermions has been calculated to be ~6.12 × 10^5^ m/s.

In conclusion, the possibilities, for switching between the high velocity properties of massless Fermions attributed to the linear dispersion relation around Dirac cone and the high on/off ratio properties associated with the sizable bandgap for a specific graphene superlattice, are carefully investigated. Upon applying uniaxial strain, such as the strains σ_z_ and σ_a_, the degeneracy between π_a_ and π_z_ bands would be removed, resulting in an energy interval E_s_ to make the Dirac cone to move. The free-standing (*P*,*Q*) (*Q* = 3*m*) PGS should have Dirac cone at Γ point according to the energy band folding analysis. Upon introducing a hexagonal antidot in the unit cell, the patterned superlattice would open a bandgap, showing a novel mechanism for bandstructure engineering as compared to the sublattice-equivalence breaking. In comparison with the defect-free PGS, the π_a_ orbitals are split. The studied *D*_6h_ defect has different effects on π_a1_- and π_a2_-type C-C bonds, making the former to contract and the latter to elongate. Similar effects are also observed for the π_z_-type C-C bonds. The energy interval between π_a1_ and π_a2_ energy bands and the value between π_z1_ and π_z2_ energy bands are same, which is referred as E_d_. For the hexagonal-antidot-patterned (*P*,*Q*) (*Q* = 3*m*) superlattice under uniaxial strain, the competition between E_s_ and E_d_ dominates whether the bandgap remains open or not. For a given superlattice, the E_d_ keeps almost unchanged while the E_s_ could be increased by enhancing the strain. Starting from the free-standing (*P*,*Q*) (*Q* = 3*m*) superlattice, which has a bandgap opened by the energy split between π_a1_ (π_z1_) and π_a2_ (π_z2_) oribitals, the bandgap decreases along with the increase of the applied uniaxial strain until it closes. Upon this critical point, the energy bands π_a1_ and π_z1_ start to cross to form new Dirac cone. So, the electronic properties of (*P*,*Q*) (*Q* = 3*m*) superlattice have been switched from the high on/off ratio properties to the high carrier velocity properties. Also, the transformation is reversible along with the strain releasing. Besides, the gap width could be continuously tuned by controlling strain, showing attractive application potentials.

## Methods

We have carried out spin-polarized first-principles calculations within the framework of density functional theory with a plane wave basis set as implemented in the Vienna *ab initio* simulation package^30^. The projector augmented-wave method was employed^31^. The exchange and correlation energy was calculated by the generalized gradient approximation with the Perdew and Wang (PW91) formulism^32^. The valence electron configurations for B, C, and N were 2s^2^2p^n^ with n = 1–3, respectively; for H, the configuration was 1s^1^. The solution of the Khon-Sham equation was calculated by an efficient matrix diagonalization technique based on a sequential band-by-band residual minimization method and a Pulay-type charge density mixing^30^. The graphene superlattice was simulated by applying a supercell with 15 Å vacuum to separate the graphene sheet with its periodic images along Z direction, which was placed in the XY plane. For the calculation of electronic structure, the cutoff energy of 400 eV was used for the planewave basis set. The *k*-point sampling was performed with the Monkhorst-Pack technique^33^. For the studies of the graphene with primitive unit cell and the smallest rectangle graphene superlattice, the integration of electronic structures was calculated by using a 15 × 15 × 1 *k*-mesh. The structural parameters of the primitive unit cell of pristine graphene were firstly optimized, which were then used to construct superlattice. In our studies, the in plane lattices of the hexagonal primitive unit cell were calculated to be 2.466 Å, which is in good agreement with the previous data^5^. For the studies of large-size orthogonal superlattices, the corresponding unit cells are 12.8 × 7.4 × 15 Å^3^ and 34.2 × 22.2 × 15 Å^3^. Thus, the *k*-meshes of 7 × 9 × 1 and 3 × 5 × 1 were used for studying their electronic properties, respectively. Correspondingly, the charge densities and the local potentials were respectively calculated by using the 128 × 72 × 160 and 336 × 224 × 160 grid meshes. The convergence of the electronic properties was set to 10^−5^ eV. In the structural optimization, all the atoms were fully relaxed until the force acting on each atom converged to 0.01 eV/Å. As to the superlattice, we have also carefully checked the effects of antidot on lattice vectors of the repeated unit. The regularly arranged antidots in fact would not change the angle between lattice vectors, which would only affect the lattice constant. Thus, the symmetry of the superlattice could be preserved, which plays an important role in determining the position of Dirac cone in reciprocal space. For the superlattices simulated in our studies, the lattice constants were carefully optimized. As for testing the accuracy of the method, we also studied the lattice constant of diamond, which was calculated to be 3.571 Å in good agreement with the experimental value of 3.567 Å^34^. In order to check the thermal stabilities of the defect patterned superlattices, we have carried out first-principles constant energy molecular dynamics (MD) simulations. The MD simulations last for 5 ps with a time step of 0.5 fs. Considering the large unit size of 34.2 × 22.2 × 10 Å^3^ used for the MD simulations and the purpose to roughly evaluate the structural stabilities, the MD simulations were carried out by using the Γ point. Due to the intensive computing loading, the energy cutoff of 300 eV was adopted for the planewave basis set. The grid size for numerical integration was chosen to be 216 × 140 × 64. The stabilities at the temperatures around 1000 and 1500 K were estimated in our studies.

## Additional Information

**How to cite this article**: Jia, T.-T. *et al*. Dirac cone move and bandgap on/off switching of graphene superlattice. *Sci. Rep*. **6**, 18869; doi: 10.1038/srep18869 (2016).

## Figures and Tables

**Table 1:** The average group velocities of massless Fermions (*v*, in the unit of 10^5^ m/s) or average effective masses of charge carriers (*m*, in the unit of the free electron mass *m*_0_) of the (6, 6) PGS and the C_12_ antidot patterned superlattice.

Material	*σ*_z_	*v*	*m*
PGS	0	8.24	—
	2%	8.15	—
	3%	8.07	—
	5%	7.90	—
C_12_	0	—	0.087
	2%	—	0.085
	3%	3.80	—
	5%	6.12	—

The corresponding energy bandstructures are studied in Fig. 6. The stretching uniaxial strain σ_z_ is applied along the zigzag edge.
